# Chromatin dynamics in pollen mother cells underpin a common scenario at the somatic-to-reproductive fate transition of both the male and female lineages in *Arabidopsis*

**DOI:** 10.3389/fpls.2015.00294

**Published:** 2015-04-28

**Authors:** Wenjing She, Célia Baroux

**Affiliations:** Department of Plant Developmental Genetics, Institute of Plant Biology and Zürich-Basel Plant Science Center, University of ZürichZürich, Switzerland

**Keywords:** *Arabidopsis*, pollen mother cells, chromatin, histone modifications, histone variants

## Abstract

Unlike animals, where the germline is established early during embryogenesis, plants set aside their reproductive lineage late in development in dedicated floral organs. The specification of pollen mother cells (PMC) committed to meiosis takes place in the sporogenous tissue in anther locules and marks the somatic-to-reproductive cell fate transition toward the male reproductive lineage. Here we show that Arabidopsis PMC differentiation is accompanied by large-scale changes in chromatin organization. This is characterized by significant increase in nuclear volume, chromatin decondensation, reduction in heterochromatin, eviction of linker histones and the H2AZ histone variant. These structural alterations are accompanied by dramatic, quantitative changes in histone modifications levels compared to that of surrounding somatic cells that do not share a sporogenic fate. All these changes are highly reminiscent of those we have formerly described in female megaspore mother cells (MMC). This indicates that chromatin reprogramming is a common underlying scenario in the somatic-to-reproductive cell fate transition in both male and female lineages.

## Introduction

In flowering plants, sexual reproduction involves the differentiation of spore mother cells (SMC) in dedicated male and female floral organs. Male SMC in the anthers give rise to haploid microspores through meiosis. The latter undergo mitosis and generate a bicellular, then tricellular male gametophyte encapsulated in the pollen grain. Foreseeably, male SMC are also referred to as pollen mother cells (PMC) or microspore mother cells in the literature. The mature male gametophyte comprises a large vegetative cell and two sperm cells. Following pollen germination, the vegetative cell grows a pollen tube delivering the sperm cells to the ovule. Each of the sperm cells will fuse with the female gametes, the egg and central cell, to generate the zygote and endosperm, respectively, following double fertilization (Maheshwari, 1950; Twell, 2011).

Cellular differentiation in the male gametophyte is accompanied by nuclear differentiation. In the mature pollen a dimorphic chromatin state is established between the vegetative cell and the sperm cells: the vegetative cell harbors a decondensed chromatin devoid of heterochromatin domains, low levels of CG DNA methylation, H3K9me2, H3K4me2, and H3K9ac histone modifications (Tanaka et al., 1998; Schoft et al., 2009; Houben et al., 2011); by contrast, the chromatin in the sperm cells is highly condensed, enriched in H3K9me2, H3K4me2, H3K9ac and harbors higher CG methylation at repeat loci (Tanaka et al., 1998; Schoft et al., 2009; Houben et al., 2011). Notably, the cells of the male gametophyte have a reduced repertoire of H3 variants, with no H3.1 isoforms and a limited number of H3.3 variants corresponding to HTR5, HTR8, HTR14 in the vegetative cell and HTR5, HTR10, HTR12 in the sperm cell (Ingouff et al., 2010). The chromatin dynamics events occurring during male gametophyte development and leading to these distinct epigenetic and transcriptional status are thought to play fundamental roles in the derepression of gametic-specific genes, keeping genome integrity in the gametes and setting epigenetic asymmetry for genome imprinting (reviewed in Gutierrez-Marcos and Dickinson, 2012; She and Baroux, 2014).

PMC are formed in a stepwise manner in the anther lobes following the division of archesporial cells (AC) located at a subepidermal position. The AC divide periclinally to give rise to a primary parietal cell and primary sporogenous cell, or PMC initials (Feng et al., 2013). In maize, a developmental analysis at high temporal and cellular resolution resolved the ontogeny of PMC leading to a model where multiple AC contribute to generate the PMC rather than a unique progenitor (Kelliher et al., 2014). In Arabidopsis, the current view is still a lineage-based model (Feng et al., 2013), which would probably deserve re-examination using similar approaches as those for resolving PMC ontogeny in maize (Kelliher et al., 2014). Nevertheless, PMC differentiation features a somatic-to-reproductive cell fate transition that, in the light of our former observations in female SMC (She et al., 2013) could possibly involve large-scale chromatin regulations contributing to establish the sporogenic competence, preparation to meiosis and post-meiotic development. Although the chromatin setup of PMC was not investigated in detail so far, there is evidence for epigenetic regulators contributing to the mitotic-to-meiotic switch. ARGONAUTE proteins are essential for epigenetic regulation based on microRNA (miRNA)- and small-interfering RNA (siRNA)-directed post-transcriptional gene silencing (PTGS) and RNA directed DNA methylation (Vaucheret, 2008). The rice AGO gene *MEIOSIS ARRESTED AT LEPTOTENE1 (MEL1)* is essential to proceed through microsporogenesis. The mutant lacking MEL1 activity arrests at early meiotic Prophase I, and notably, some PMC in the mutant that arrested at leptotene or zygotene stage showed decreased H3K9me2 intensity and altered chromatin organization at the nucleolus organizing region (NOR). This suggests global chromatin mechanisms necessary for the meiotic competence of PMC (Nonomura et al., 2007). Furthermore, the observations of large nuclei with decondensed chromatin in early drawings of primary sporogenous cells in anthers in different species (Pozner, 2001) suggest that large-scale chromatin dynamics may take place in differentiating PMC. To resolve this, we quantitatively analyzed nuclear organization and chromatin modifications in differentiating PMC and found that, similar to that in female SMC, PMC undergo drastic chromatin reorganization, suggesting a common event during the somatic-to-reproductive cell fate transition in both genders in plants.

## Materials and methods

### Plant material and growth conditions

Young anthers were collected from *Arabidopsis* plants grown under long-day condition (16 h light/8 h dark) at 18–20°C in a plant growth chamber. H2A.Z-GFP is pHTA11:HTA11-GFP (Kumar and Wigge, 2010). H1.1-GFP and H1.2-GFP lines were from the same seed stock as that described in She et al. (2013).

### Immunostaining in whole-mount anthers

Immunostaining was performed as described for whole-mount ovule primordium immunodetection (She et al., 2013, 2014) with minor modifications for anthers: young anthers were fixed in 1% formaldehyde and 10% DMSO in PBS-Tween (0.1%), followed by dissection and embedding of the anthers in 5% acrylamide pads on microscope slides. Tissues were then processed by clarification (methanol/xylene), cell wall digestion, permeabilization, and 5% BSA blocking (40 min to 1 h). After that, the samples were incubated with the primary antibody for 12–14 h and then the secondary antibody for 24–48 h at 4°C. The tissues were counterstained with propidium iodide and mounted in Prolong Gold (Invitrogen). The primary antibodies against H1, H3K27me1, H3K27me3, H3K4me2, and H3K4me3 as well as the secondary antibodies are all diluted by 1:200. The primary antibodies are: anti-H1 (Agrisera, AS11 1801), anti-H3K27me1 (Abcam 07-448), anti-H3K27me3 (Active motif, 39155), anti-H3K4me2 (Abcam ab32356), anti-H3K4me3 (Abcam, ab8580). Immunostaining has been standardized as described (She et al., 2014) with respect to (i) antibody dilution: a dilution series followed by quantification allows to determine the linear phase of detection, (ii) a negative control: immunostaining without primary antibody allows to test for background signal, (iii) a positive control: immunostaining against a constitutive component, for instance H3 (She et al., 2013), or H3K4me2 in this study, allows to test for homogenous accessibility of the antibodies throughout the tissue.

### Image acquisition

Image series of fluorescent signals in whole-mount embedded anthers were acquired by confocal laser-scanning microscopy (CLSM, SP5-R, Leica Microsystems, Germany) using a 63× GLY lens (glycerol immersion, NA 1.4). Images were recorded sequentially for the antibody and DNA fluorescence channels respectively and the volumes were sampled based on the Nyquist rate (2× oversampling). Settings for the parameters including zoom factor, image geometry, voxel size, scanning speed, and averaging were identical for the image series recorded for the immunostaining experiment where the same primary antibody was applied.

For GFP-tagged histone reporter lines, anthers were dissected from young flower buds in 0.5× MS (Murashige and Skoog) and fluorescent signals were recorded with CLSM as before (SP5-R system, 63× GLY lens) with excitation = 488 nm, emission = 500–520 nm under conservative settings (low laser power, low gain, high scanning speed, 12 frame averaging).

### Quantitative analyses

Fluorescent signals (GFP, antibody staining, DNA staining) were measured in 3D reconstructions of CSLM images of immunostained, whole-mount anthers using the Imaris software (Bitplane, CH), as described in a former study (She et al., 2013) and in an online tutorial (http://www.bitplane.com/learning/quantification-of-chromatin-modifications-in-whole-mount-plant-tissue-tutorial). In brief, (i) PMC and surrounding anther nuclei were segmented using the Manual Contour Surface tool, (ii) fluorescent signals from the antibody (Ab) channel and the propidium iodide (PI) were measured as the intensity sum of voxels per channel (Surface Statistics), respectively, (iii) the levels of H3 modifications were expressed relative to the DNA levels (relative Fluorescence Intensity, FI) and correspond to the ratio of Ab/PI intensity sums, (iv) the average Ab/PI ratio in PMC was expressed relative to the surrounding cells (FI set to 100%).

Nuclear volumes were measured in 3D reconstructions of CSLM images of PI-stained whole-mount anthers using the Imaris software (Bitplane, CH). The diameter was measured in the x, y and z dimensions in the Section View mode and the nuclear volume was calculated as V = $\frac{\pi }{6}$*xyz*.

The relative heterochromatin fraction (RHF) and the number of chromocenters were measured using ImageJ as previously described (She et al., 2013) on intensity sum projections from 3D images encompassing (non-overlapping) MMC and nucellus nuclei. The RHF consisted in the sum of intensity signals in chromocenters (contours defined manually) expressed as a percentage of the total nuclear fluorescence intensity.

Differences in replicate quantifications (the number of observation, n, is indicated in each graph) were assessed using a two-tailed Welch's *t*-test. Graphs show error bars corresponding to the standard error of the mean.

## Results

### Chromatin decondensation in *Arabidopsis* PMC

**Figure 1:**
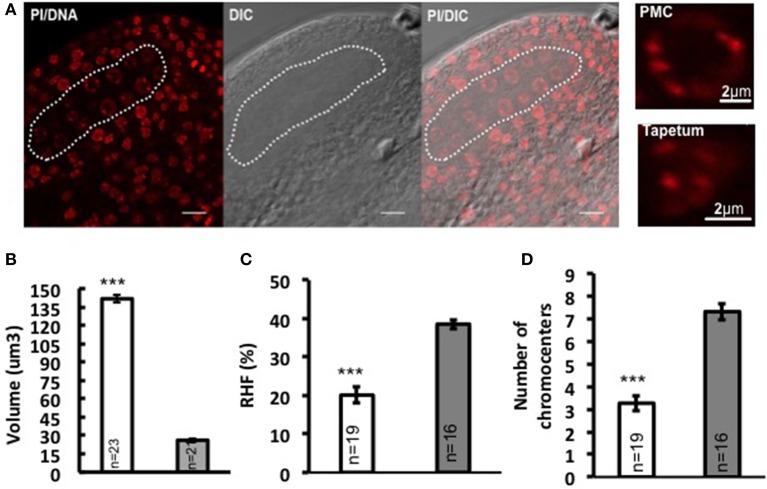
**Nuclear reorganization in differentiating PMC. (A)** Specification of PMC in the early Arabidopsis anther locule. A representative image of an anther locule stained for DNA is shown (Propidium iodide, PI, red), differential interference contrast, and the DIC image overlaid with PI counterstaining. The male sporangium forming the PMC is indicated by a white dotted line. Scale bar: 10 μm. Compared to surrounding somatic cells like tapetum nuclei, PMC nuclei are marked by significantly enlarged nuclear and nucleolar size, as is shown by the images on the right panel. Comparison of nuclear volume **(B)**, heterochromatin content (**C**, RHF, relative heterochromatin fraction) and chromocenter number **(D)** between PMC (shown in white dotted line) and epidermal cells was based on quantitative analyses on 3D reconstructed whole-mount, embedded young anthers. PMC, white bars; Epidermal cells, gray bars. The number of nuclei analyzed was given in each bar (n). Differences between chromatin of PMC and epidermal cells in replicate quantitative measurements were assessed using a two-tailed Welch's *t*-test (^***^P < 0.001). Error bars represent the standard deviation to the mean (s.e.m).

In flowering plants, the development of the male reproductive lineage is initiated with the differentiation of PMC in the early anther locule. In *Arabidopsis*, a sub-epidermal somatic cell in the sporangium forms the archesporial cell that then divides to form the primary parietal cell toward the exterior and the primary sporogenous cell toward the interior. The primary parietal cell will produce four layers of cells comprising the anther wall after mitosis, while the primary sporogenous cell divides to give rise to two layers of PMC (Pozner, 2001). Consistent with former observations (Pozner, 2001), we observed changes in nuclear morphology of differentiating PMC, marked by enlargement of nuclei and nucleoli (Figure 1A). Quantitative analyses on series of images of whole-mount, embedded young anthers revealed a 5-fold increase in nuclear volume of PMC (141.751 μm^3^ ± 7.92, *n* = 23) compared to that of the epidermal cells in the anther wall (26.101 μm^3^ ± 2.23, *n* = 21) (Figure 1B; Supplementary Material Table S1). This is accompanied by a 47.9% decrease in heterochromatin content (Figure 1C; Supplementary Material Table S1) and reduction in the mean number of distinct chromocenters from ca. 7 down to 3 in average in PMC (Figure 1D). Thus PMC harbor a distinct nuclear organization compared to that of somatic cells of the anther walls.

### Alterations of histone variant composition in Arabidopsis PMC

**Figure 2:**
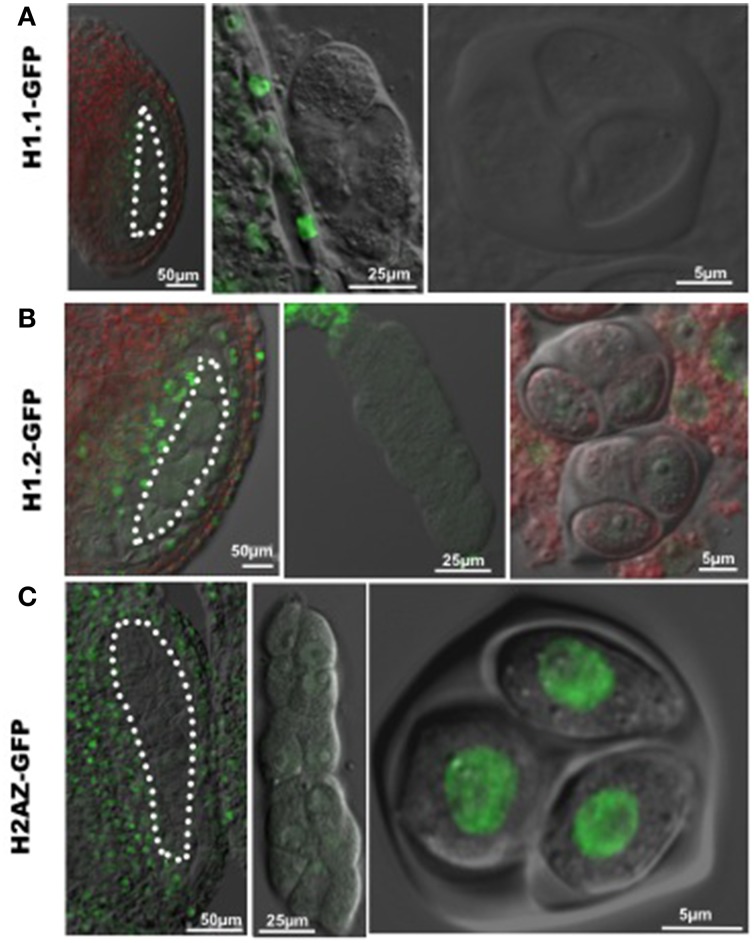
**Dynamic nuclear distribution of GFP-tagged histone variants in developing anthers. (A,B)** Expression of GFP-tagged linker histone variants H1.1 and H1.2 in the developing anther. GFP fluorescence was hardly detectable in differentiating PMC (images at the left and middle panels) and the tetrad (images at the right panel), which indicates an eviction of linker histone H1 in PMC and the tetrad. **(C)** Expression of the GFP-tagged histone variant H2A.Z in the developing anther. GFP-tagged H2A.Z is well expressed in surrounding somatic cells in the anthers, but absent in PMC (images at the left and middle panels). GFP-H2A.Z is restored after meiosis in the tetrad (the image at the right panel). PMC are marked by white dotted line. Green, GFP fluorescence.

The increase in nuclear volume, and decrease of heterochromatin content indicate chromatin decondensation in PMC similar to that in female SMC (megaspore mother cell, MMC). Linker histone H1 is required for chromatin compaction (Hood and Galas, 2003). The *Arabidopsis* genome encodes three canonical H1 variants, including H1.1, H1.2, H1.3, with H1.1 and H1.2 being the most abundantly expressed variants during plant development (Wierzbicki and Jerzmanowski, 2005). To investigate whether chromatin decondensation in PMC correlates with changes of linker H1, we analyzed H1.1 and H1.2 expression of GFP-tagged variants in the developing anther, and found a drastic reduction of fluorescence in PMC (Figures 2A,B), confirmed in immunostaining using an antibody against plant H1s (Supplementary material Figure S1). This may result from either eviction of H1 variants in the PMC chromatin like in the MMC, or possibly a transcriptional downregulation after the sporogenous initials. In addition, similarly to the situation in MMC, we found a reduction in PMC of a GFP-tagged H2A.Z, a histone variant antagonizing DNA methylation and chromatin compaction (reviewed in March-Diaz and Reyes, 2009). However, strong fluorescent signals from GFP-tagged H2A.Z were detected in the tetrad, suggesting that H2A.Z is reloaded at or shortly after meiosis (Figure 2C).

### Establishment of a distinct epigenetic status in PMC compared to surrounding somatic cells

**Figure 3:**
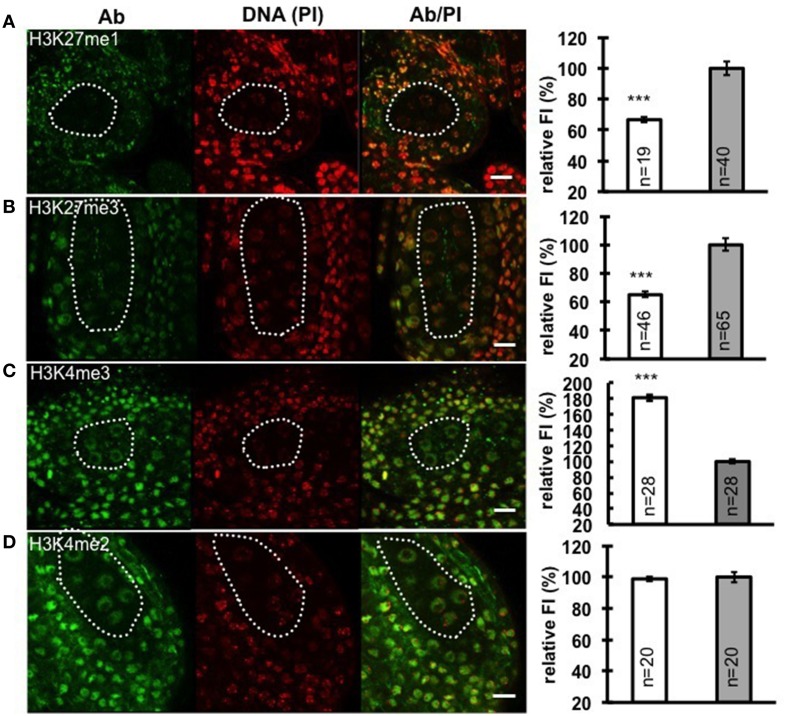
**Immunodetection and quantification of whole-mount anther locules reveal distinct patterns of chromatin modification in PMC**. Global levels of H3K27me1 **(A)**, H3K27me3 **(B)**, H3K4me3 **(C)**, and H3K4me2 **(D)** were measured in PMC nuclei (white dotted line) and surrounding somatic cells. The fluorescence intensity (FI) corresponding to immune-signals intensity (Ab) over propidium iodide intensity (PI) in PMC nuclei is expressed relative to the corresponding Ab/PI ratio in surrounding cells. The quantifications were done in 3-dimensional reconstructions of confocal images of whole-mount embedded anthers using Imaris (see Methods, She et al., 2013, 2014). Representative images are shown for the antibody (Ab, green), DNA (Propidium iodide, PI, red), and the Ab fluorescence image overlaid with PI counterstaining (Ab/PI). Scale bar: 10 μm. PMC, white bars; Surrounding somatic cells, gray bars. The number of nuclei analyzed was given in each bar (n). Differences between chromatin of PMC and surrounding somatic cells were assessed using a two-tailed Welch's *t*-test (^***^P < 0.001). Error bars represent the standard deviation to the mean (s.e.m).

Chromatin decondensation and reduction of H1 levels suggest a transcriptionally permissive chromatin landscape is established in PMC, a chromatin state likely reflected by specific post-translational modifications of histones. To analyze the distribution of histone modifications in PMC, we focused on histone marks associated with either repressive euchromatin regions (H3K27me3), or repressive heterochromatin regions (H3K27me1), as well as permissive euchromatin regions (H3K4me2 and H3K4me3). We performed immunostaining with antibodies against these above-mentioned histone marks on whole-mount embedded young anthers and quantified the antibody signal levels relative to the DNA content in PMC and surrounding somatic cells of the anther wall. Notably, we measured a 33% decrease of H3K27me1 signals (Figure 3A; Supplementary Material Table S2) and 35% reduction of H3K27me3 (Figure 3B; Supplementary Material Table S2). The permissive mark H3K4me3 is characterized by 1.8-fold increase in PMC compared to that in somatic cells in the anther walls (Figure 3C; Supplementary Material Table S2). However, the level of H3K4me2 in PMC was constant, compared to that in surrounding somatic cells (Figure 3D; Supplementary Material Table S2). The distinct chromatin modification pattern established in PMC is similar to that we observed in the MMC (She et al., 2013).

Collectively, PMC differentiation, as in female SMC, is marked by global changes of histone modifications. Removal of the repressive marks including H3K27me1 and H3K27me3, and enrichment of the permissive mark H3K4me3, suggest the establishment of a permissive chromatin that may entail an active transcriptional state in PMC.

## Discussion

The work described here illustrates that PMC differentiation is characterized by global dynamic changes of nucleosomal organization and chromatin modifications in *Arabidopsis* PMC. A distinct chromatin state is established in differentiating PMC, with enlarged nuclear volume, reduction of heterochromatin, removal of histone variants, and dramatic changes in the pattern of histone modifications compared to that in somatic cells that comprise the anther wall.

### Chromatin reorganization in PMC is similar to that of MMC

Interestingly, chromatin reorganization in *Arabidopsis* PMC is similar to most of the events occurring in the MMC, which suggests a conserved scenario of chromatin dynamics in plant SMC that may be required for the somatic-to-reproductive cell fate transition (She et al., 2013). As in the MMC, eviction of linker histone H1 also occurs in PMC. Possibly, a CDK2-mediated phosphorylation of H1 may contribute to destabilizing H1 binding to the chromatin as shown in wheat (Contreras et al., 2003; Greer et al., 2012). H1 eviction is consistent with chromatin decondensation in PMC, which is accompanied by nuclear swelling and reduction of heterochromatin content, a nuclear phenotype reminiscent of mutant lacking DDM1 activity (Soppe et al., 2002; Slotkin et al., 2009). This dynamic remodeling of chromatin composition is also consistent, and may possibly be mechanistically linked with the rapid turnover of the centromeric H3 variant in PMC (Ravi et al., 2011; Schubert et al., 2014). Furthermore, the reduction in heterochromatin domains correlates with the reduction of the associated H3K27me1. Whether this results from a passive dilution upon chromatin replication or from active removal remains to be determined. Besides, in euchromatin, the reduction in the repressive mark H3K27me3, as well as the increase of the permissive mark H3K4me3 in PMC suggests a distinct epigenetic landscape that mimics that of the MMC chromatin. However, in contrast with MMC chromatin harboring depleted levels of H3K4me2 (She et al., 2013), H3K4me2 levels were similar between PMC and the surrounding anther tissue.

Chromatin dynamics in the MMC takes place during a long meiotic S phase, suggesting that at least some of the depletion events may result from a passive dilution of modified histones coupled to DNA chromatin replication. Similarly, a long meiotic S-phase also takes place in PMC (Bennett, 1971; Armstrong et al., 2003). We detected up to 1.7-fold increase in DNA content in PMC compared to the surrounding somatic, tapetal cells that seem to undergo replication after PMC differentiation (Stronghill et al., 2014), suggesting a near complete DNA replication at the (post-mitotic) stage where we performed our quantitative chromatin analyses (Supplementary Material Figure S2). Interestingly, a reduction of the absolute level of H3K27me1 but not of H3K27me3 was detected in PMC (Supplementary Material Figures S3A,B). This suggests that H3K27me1 may be actively demethylated in PMC, while the apparent reduction of H3K27me3 may be a result of passive dilution due to *de novo* incorporation of non-modified histones. Active chromatin modifying mechanisms are expected to take place to achieve H1 and H2A.Z eviction, but also to increase H3K4 methylation in PMC (here) as well as in MMC (She et al., 2013). Whether active deposition of H3K4me3 in PMC is catalyzed by the specific SET-domain proteins ATX1 (Alvarez-Venegas et al., 2003) and SDG2 (Guo et al., 2010) remains to be determined. Accordingly, the developmental arrest of microspores in the *sdg2* mutant (Berr et al., 2010; Guo et al., 2010) favors the idea of an implication of SDG2 in setting a novel epigenetic landscape in PMC.

### Possible roles of chromatin reorganization in PMC

Differentiation of PMC is followed by meiotic execution and entails the establishment of a novel developmental competence that achieves the male gametophyte. Profiling of male meiocytes from various plant species indicates a massive transcriptome alteration with activation of a large number of both meiotic-specific and non-meiotic specific genes Reviewed by Zhou and Pawlowski (2014). It was shown recently that meiotic genes are activated at an early stage of PMC specification prior to the meiotic S-phase in maize (Kelliher and Walbot, 2014). It is thus tempting to propose that chromatin dynamics in PMC enables reprogramming of the transcriptional, and likely epigenetic landscape necessary for meiotic gene activation and preparation for post-meiotic development. However, functional analyses, for instance using PMC specific mutations affecting distinct aspects of chromatin dynamics are necessary to validate this hypothesis.

Meiotic functions of chromatin dynamics may be formulated with the question whether H1 eviction, chromatin decondensation, and H3K4 methylation directly promote meiotic gene activation, reset the chromatin structure for meiotic processes, or both. For instance, H3K4me3, DNA methylation and nucleosome remodeling are instructive for crossing-over and double-strand-break repair process involved in recombination (Yelina et al., 2012; Choi et al., 2013). Chromatin dynamics is biphasic in PMC with an early eviction of H1 and H2A.Z, yet reloading at the onset of meiotic Prophase I (Supplementary Material Figure S4). This suggests two distinct roles for H1, reloading being most likely underlying structural requirements for chromatin condensation during meiotic Prophase I. Deficiency of linker histone variants results in dramatic chromosomal aberrations during male meiosis in tobacco, including the appearance of micronuclei or parts of chromosomes scattered throughout the cytoplasm. This indicates that linker histone is essential for meiotic progression (Prymakowska-Bosak et al., 1999). In wheat, failure in chromatin reorganization at the onset of meiosis leads to compromised homologs pairing (Colas et al., 2008).

Whether chromatin dynamics in PMC underlies the establishment of post-meiotic developmental competence should be addressed with a temporal fine resolution of induced perturbations in stable mutants with sporophytic and sporogenic effects. In the *sdg2* mutant, impaired in SDG2-mediated H3K4 trimethylase activity, a large proportion of meiocytes undergo meiosis yet do not proceed normally through gametophyte development. This might suggest a role of *SDG2* in PMC to establish a post-meiotic developmental competence in the male reproductive lineage, similarly to the situation in MMC (She et al., 2013). In this mutant, however, developmental defects in tapetal and sporogenous cell differentiation mask potential PMC-specific functions of *SDG2*.

## Conclusion and perspectives

We report here large-scale chromatin dynamics in PMC affecting heterochromatin organization and the distribution of chromatin modifications reminiscent of those in MMC, thereby underpinning a common basis for the somatic-to-reproductive cell fate transition in the male and female lineages of Arabidopsis. This reveals unexpected remodeling of the epigenetic landscape in SMC, i.e., prior to meiosis, and in addition to post-meiotic epigenetic reprogramming in the gametophytes reviewed by She and Baroux (2014) and Kawashima and Berger (2014). Future investigation should focus on the functional elucidation of chromatin dynamics in SMC, particularly with respect to reprogramming toward pluri- or totipotency, maintenance of genome integrity, epiallelic variation and regulation of imprinting during plant sexual reproduction. For this aim, challenging technical issues remain to be solved in order to provide a cell-specific reading of the epigenome and chromatin states at the gene-level in SMC.

### Conflict of interest statement

The authors declare that the research was conducted in the absence of any commercial or financial relationships that could be construed as a potential conflict of interest.
