# Supplementary material for: Chromatin dynamics in pollen mother cells underpin a common scenario at the somatic-to-reproductive fate transition of both the male and female lineages in Arabidopsis
Source: Front Plant Sci. 2015 Apr 28;6:294. doi: 10.3389/fpls.2015.00294 (PMC4411972; doi:10.3389/fpls.2015.00294)
Supplement: Supplementary file 1 [file Table1.DOCX]

**Table S1. Detailed quantification of nuclear volume and heterochromatin content in PMCs and epidermal cells during pollen development**

|  |  | |  | |  | |  | |  | |  | |  | |  | |
| --- | --- | --- | --- | --- | --- | --- | --- | --- | --- | --- | --- | --- | --- | --- | --- | --- |
|  | | **PMC** | | | | | | **Epidermal cells** | | | | | |  | |  |
|  | | **average** | | **s.d.** | | **n** | | **average** | | **s.d.** | | **n** | |  | |  |
| **Nuclear Volume (μm^3^)** | | 141.751 | | ± 7.92 | | (n=23) | | 26.107 | | ± 2.23 | | (n=21) | | P<0.0001 | |  |
|  | |  | |  | |  | |  | |  | |  | |  | |  |
| **RHF** | | 20.101 | | ± 4.38 | | (n=19) | | 38.567 | | ± 2.39 | | (n=16) | | P<0.0001 | |  |

The Relative Heterochromatin Fraction (RHF) is calculated as a % ratio of fluorescence intensity (propidium iodide) in heterochromatic foci over intensity in the surrounding somatic cells of the anter wall. s.d., standard deviation (note that the graphs show the standard error to mean= s.d/√n). P-value: Welch'st-test (2 tails).
