# Supplementary material for: Chromatin dynamics in pollen mother cells underpin a common scenario at the somatic-to-reproductive fate transition of both the male and female lineages in Arabidopsis
Source: Front Plant Sci. 2015 Apr 28;6:294. doi: 10.3389/fpls.2015.00294 (PMC4411972; doi:10.3389/fpls.2015.00294)
Supplement: Supplementary file 2 [file Table2.DOCX]

**Table S2: Detailed quantifications of relative nuclear immunostaining in PMCs and surrounding somatic cells of the anther wall**

| (a) Antibody signal over propidium iodide signal, relative to the surrounding somatic cells | | | | | | | | | | | |
| --- | --- | --- | --- | --- | --- | --- | --- | --- | --- | --- | --- |
|  | | |  | |  | |  |  |  |  |  |
|  | | | **PMCs** | | | | | **somatic cells** | | |  |
|  | | | **%** | | **s.d** | | **n** | **%** | **s.d** | **n** |  |
| **H3K27me1** | | | 66.65 | | ± 3.428 | | (n= 19) | 100 | ± 14.92 | (n= 40) | P= 1.61E-08 |
| **H3K27me3** | | | 65.07 | | ± 6.91 | | (n= 46) | 100 | ± 17.15 | (n= 65) | P= 6.77E-11 |
| **H3K4me3** | | | 181.27 | | ± 10.66 | | (n= 28) | 100 | ± 9.89 | (n= 28) | P= 1.41E-20 |
| **H3K4me2** | | | 98.8 | | ± 3.623 | | (n= 20) | 100 | ± 7.592 | (n= 20) | P= 0.753693 |
|  | | |  | |  | |  |  |  |  |  |
| (b) Antibody signal over propidium iodide signal- absolute ratios in the surrounding somatic cells | | | | | | | | | | | |
|  | | |  | |  | |  |  |  |  |  |
|  | | | **somatic cells** | | | | |  |  |  |  |
|  | | | **Ab/PI** | | **s.d** | | **n** |  |  |  |  |
| **H3K27me1** | | | 1.19267 | | ± 0.18 | | (n= 40) |  |  |  |  |
| **H3K27me3** | | | 1.23707 | | ± 0.21 | | (n= 65) |  |  |  |  |
| **H3K4me3** | | | 1.66391 | | ± 0.16 | | (n= 28) |  |  |  |  |
| **H3K4me2** | | | 2.04093 | | ± 0.15 | | (n= 20) |  |  |  |  |
|  |  |  | |  | |  |  |  |  |  |  |

The relative immunostaining signals are calculated as fluorescence intensity ratios of Antibody (Ab) signals over Propidium Iodide (PI) signals. (a) The ratios in somatic cells are averaged across n samples and set as 100%. Ab/PI ratio in PMCs relative to that in somatic cells. (b) absolute ratios in somatic cells. s.d., standard deviation. (note that the graphs show the standard error to mean= s.d/√n). P-value: Welch's t-test (2 tails).
