# Supplementary material for: Chromatin dynamics in pollen mother cells underpin a common scenario at the somatic-to-reproductive fate transition of both the male and female lineages in Arabidopsis
Source: Front Plant Sci. 2015 Apr 28;6:294. doi: 10.3389/fpls.2015.00294 (PMC4411972; doi:10.3389/fpls.2015.00294)
Supplement: Supplementary file 3 [file Image1.PDF]

SUPPLEMENTARY FIGURES

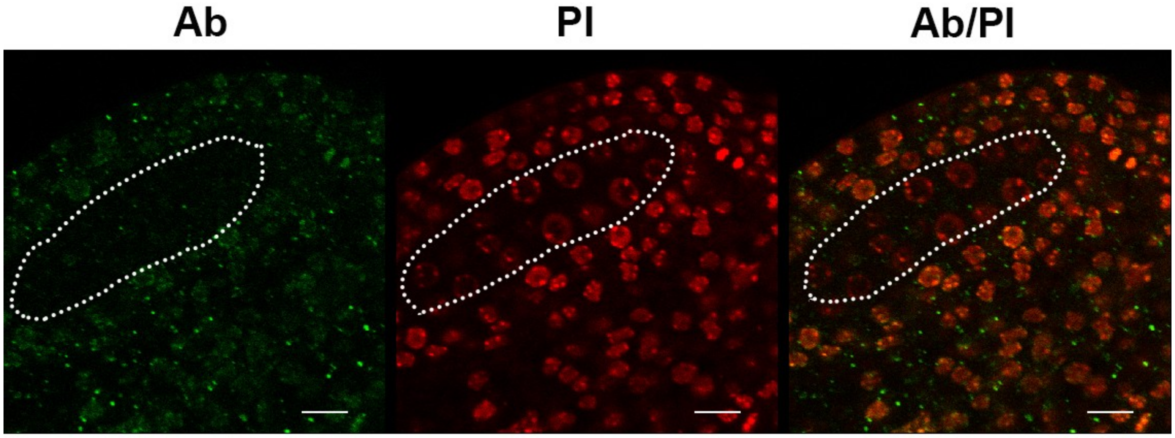

Figure S1. Dramatic reduction of linker histone H1 in PMCs, as confirmed by whole-mount immunostaining in the anther locule with a specific antibody against H1

H1 is almost undetectable in PMCs (dashed line). Representative images are shown for the antibody (Ab), DNA (Propidium iodide, PI) and antibody signal overlaid with the DNA signal (Ab/PI).

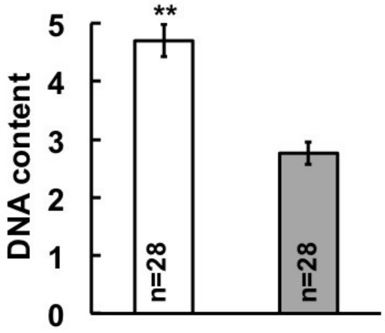

Figure S2. A meiotic S-phase was detected in PMCs.

Quantification of the DNA content in PMCs compared with that of the surrounding somatic cells comprising the anther suggests replication of DNA in PMCs. The average DNA fluorescence intensity was shown for PMCs and somatic cells respectively in the bar chart; the number of nuclei analyzed were given in each bar (n). Error bars indicate s.e.m. PMCs, the white bar; Somatic cells, the grey bar.

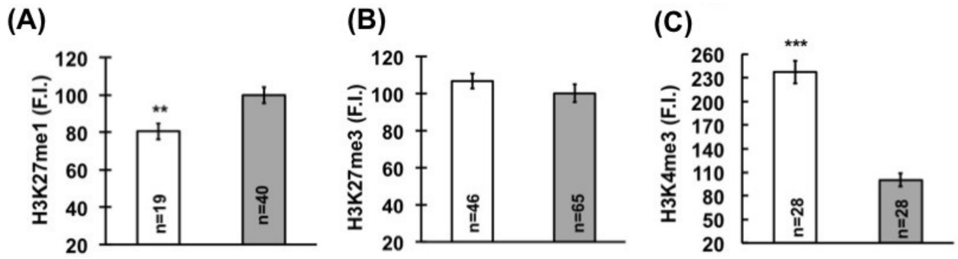

Figure S3. Quantification of the absolute levels of histone modifications in PMCs indicates both active and passive ways contribute to dynamic changes of PMCs chromatin

(A) Decreased levels of H3K27me1 in wild-type PMCs relative to the surrounding somatic cells is visible in absolute values of immunostaining signals suggesting both a replication-coupled passive dilution and a probable active demethylation process. **B**. H3K27me3 levels remain constant in PMCs compared to that of the somatic cells from the anther wall, which indicates a replication-dependent passive dilution of H3K27me3 in PMCs. **C.** The absolute level of H3K4me3 in PMCs is increased relative to the somatic cells comprising the anther wall, suggesting an active methylation of H3K4 in PMCs.

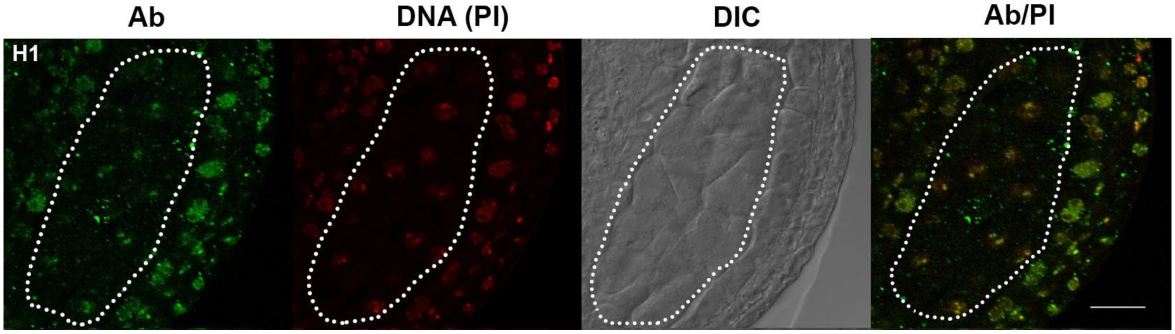

Figure S4. Reloding of H1 into PMCs at the onset of meiosis, as revealed by whole-mount immunostaining in the anther locule with a specific antibody against H1

In prophase I, immunostaining signals of H1 are visible in PMCs, suggesting reloading of H1 into PMCs at the onset of meiosis. Representative images are shown for the antibody (Ab), DNA (Propidium iodide, PI), transmission light (DIC, grey) and antibody signal overlaid with the DNA signal (Ab/PI). Dotted contours: PMCs in prophase I. Scale Bar: 15 µm.
